# The Penal Powers of the General Medical Council

**Published:** 1916-06-10

**Authors:** 


### THE PENAL POWERS OF THE GENERAL MEDICAL COUNCIL.

The General Medical Council does not, as is so commonly supposed, exist to protect the interests of the medical profession. Its motive is the welfare of the public. For this purpose it was created by Act of Parliament, and by this standard its operations have to be judged. By the construction of the Medical Register the Council enables everyone to distinguish the legally qualified medical practitioner from persons who profess to practise medicine without this equipment; and by fixing the conditions which govern admission to the Register it maintains the adequacy of medical education and attainment. Clearly such a scheme is an important concern for the public. Equally clear is it that when a practitioner has once gained admission to the Register his subsequent retention on the list shall depend on worthy conduct. The standard of worthiness must not be a freakish or unpractical one, but it must be sufficient to secure that ill-doers do not escape the appropriate penalties. Here, again, anxiety for the public welfare is the operating influence. It is for this end that the Council is endowed with the power to summon before it any accused practitioner, and, if justice demands such a step, to remove his name from the official list. The penalty is a severe one, and means not only loss of status but often, also, loss of livelihood.

There is one point concerned in the penal powers of the Council which has often excited comment. It is that in order to remove a practitioner's name from the Register the Council must find him guilty of "infamous conduct in a professional respect." This is the statutory phrase, and it exists, therefore, as a technical necessity. Whatever be the nature of the offence, it has to be dressed in the official term. Now, infamous conduct will sound to everyone appropriate enough to a practitioner found guilty of some serious crime and condemned by the law of the land to penal servitude or imprisonment. But it is apt to appear excessive when applied to proceedings in which a moral failure is not particularly conspicuous—say, for example, the practice of self-advertising or the administration of anæsthetics to the patients of an unqualified practitioner. We are not defending such actions. Nor do we say that they ought not to mean the removal of a practitioner's name from the Register. But we do say that to the public ear the application to them of the term "infamous" sounds in excess of the facts. Hence the decree of the Council in such cases, instead of possessing the appearance of a cold and impartial justice, is apt to take on the quality of prejudice and even of professional revenge. This is an unhappy conclusion to support, and it has, of course, no real basis in point of fact. But, so long as the term "infamous" must needs appear in the judgment of the Council, whatever be the nature of the offence, some not unfair-minded people will be likely to take the view that something other than the public interest is the animating motive.

